# Civilian Fatalities Resulting From an Accidental Explosion: An Autopsy Report of Two Cases

**DOI:** 10.7759/cureus.83258

**Published:** 2025-04-30

**Authors:** Sanjeet Kumar, Kumar Shubhendu, Ankur Chaudhary, Anand Kumar

**Affiliations:** 1 Department of Forensic Medicine and Toxicology, Shaheed Nirmal Mahto Medical College, Dhanbad, IND; 2 Department of Forensic Medicine and Toxicology, Rajendra Institute of Medical Sciences, Ranchi, IND

**Keywords:** accidental bomb explosion, blast injury, civilian fatalities, explosion, medico-legal autopsy

## Abstract

A blast injury represents a complex category of physical trauma that results from either direct or indirect exposure to an explosive event, often culminating in untimely deaths and exerting pressure on healthcare systems. The range of blast injuries includes visceral, extremity, and ocular trauma, varying intensities of thermal injuries, as well as auditory dysfunctions induced by the blast. The present case report details two deaths that occurred due to an accidental bomb explosion in a vegetable market.

## Introduction

Bomb explosion injuries constitute a multifaceted and severe form of trauma resulting from detonations in military and civilian contexts, often linked to terrorism and unexpected incidents. The categorization of these injuries includes primary, secondary, tertiary, quaternary, and quinary types, each defined by distinct mechanisms and outcomes [1-3]. Primary blast injuries occur from rapid atmospheric pressure changes due to the blast wave, predominantly affecting gas-filled organs like the lungs [4]. In contrast, secondary blast injuries arise from shrapnel and debris, leading to penetrating injuries, fractures, and traumatic amputations, with a notable incidence of extremity injuries [5]. Tertiary blast injuries result from individuals striking solid objects, causing blunt trauma, including a spectrum of traumatic brain injuries from mild concussions to severe penetrations [4,6]. Quaternary blast injuries encompass various other explosion-related injuries, such as burns, crush injuries, and toxic inhalation, contributing to the total trauma load [7]. Finally, quinary blast injuries pertain to the clinical ramifications of environmental pollutants following detonation, involving chemical, biological, and radiological factors [1].

Bomb blast injuries continue to pose a significant public health and forensic concern in India, with sociodemographic studies consistently revealing a predominance of male victims. This pattern is particularly evident in states like Maharashtra and regions such as Hyderabad, where male casualties significantly outnumber their female counterparts. Additionally, the sociodemographic analysis highlights that most victims originate from urban areas, notably Mumbai, which experiences a higher incidence of bomb blast casualties compared to rural regions [8,9]. The injuries sustained are diverse, including penetrating wounds, fractures, and tympanic membrane perforations, which may lead to considerable disabilities [9]. The critical nature of these injuries often necessitates prompt and extensive medical intervention, with severe injuries correlated to elevated mortality rates, particularly in cases of thoracoabdominal trauma [7]. Sociodemographic data from Imphal, Manipur, reveal analogous trends, where ballistic injuries resulted in deaths primarily due to multiple injuries and head trauma, affecting mostly men in the 31-40-year age group [10].

The escalating occurrence of terrorist bombings on a global scale underscores the pressing necessity for comprehensive international security protocols and improved healthcare readiness. Prompt emergency interventions and proficient hospital treatment play a pivotal role in alleviating casualties and minimizing enduring disabilities, as demonstrated by case studies from events like the Hyderabad explosions [9,11]. In light of the intricate and frequently grave nature of blast-induced injuries, a multidisciplinary strategy, integrating medical, surgical, and psychological care, is crucial to enhance recovery outcomes and diminish the long-term ramifications for affected individuals and their communities.

The present case study examines the autopsy findings of two individuals who died following the accidental detonation of an explosive device in a marketplace primarily engaged in vegetable trade. A total of five female individuals, primarily consisting of vendors operating along the roadside, and one male individual, who was allegedly transporting the explosive device on his motorcycle, suffered significant injuries. Among the six who suffered injuries, two unfortunately could not survive the medical efforts and later had autopsies conducted at the Department of Forensic Medicine and Toxicology, Rajendra Institute of Medical Sciences, Ranchi, Jharkhand, India. This report aims to underscore the forensic significance and public health implications of accidental blast injuries in civilian environments, particularly in the Indian context where such events are uncommon yet devastating. By presenting these two cases, we highlight the critical role of forensic evaluation in understanding the injury mechanisms, contributing factors, and preventive strategies for blast-related trauma outside of conflict zones.

## Case presentation

Case 1

The 27-year-old woman exhibited an average physique, marked by widespread rigor mortis, slight abdominal distension, a surgical incision on the left lower abdomen indicative of drainage tube placement, singed scalp and body hairs, and diffuse tattooing across various body regions including the right side of the face, shoulders, right arm, breasts, right thigh, and adjoining right knee. First- and second-degree infected burn areas involved the face, right clavicular region, fronto-medial aspect of the right arm, posterolateral aspect of the right forearm, upper part of the posterior aspect of the right arm, left fronto-lateral aspect of the middle part of the neck, left upper limb including the dorsal aspect of the left palm, fronto-lateral aspect of the lower part of the left breast and adjoining fronto-lateral aspect of the abdomen, and fronto-medial aspect of the lower part of the left thigh and adjoining fronto-medial aspect of the left leg.

**Figure 1:**
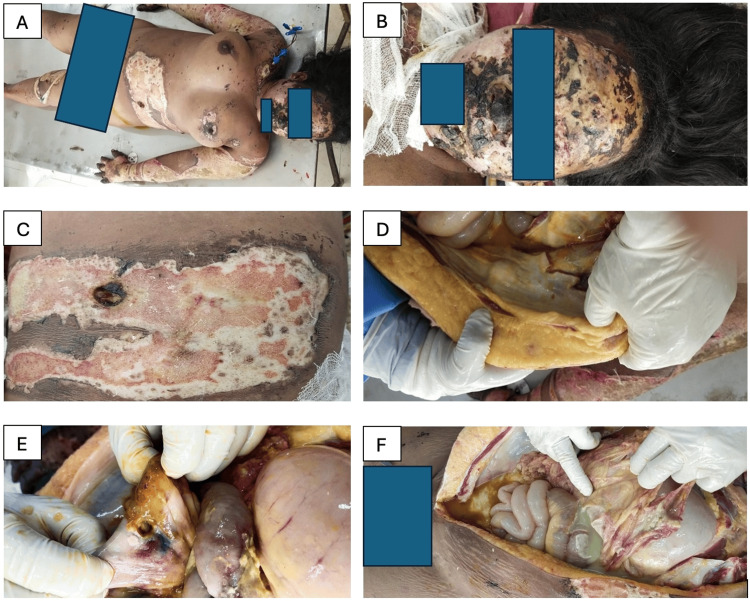
Autopsy photographs illustrating key findings in Case 1 A: Diffuse areas of tattooing and burns B: Areas of tattooing and burns over the face associated with singeing of hairs C: Lacerated wound over the left anterior aspect of the abdomen associated with skin charring D: Inner abdominal wall corresponding to the lacerated wound depicted in Figure 1C showing the wound not to be cavity deep E: Perforation of the small intestine (primary blast injury) F: Adhesion of abdominal viscera with the presence of purulent fluid intermixed with fecal matter

Two soft tissue-deep lacerated wounds were observed in the examination, with one having a diameter of 2 cm located on the left anterior aspect of the upper abdomen. This particular wound was surrounded by skin charring and subcutaneous tissue damage spanning an area ranging from 1 cm to 2 cm around the laceration, with concomitant singeing of body hairs. The second wound, measuring 7×0.5 cm, was identified on the anterior aspect of the right knee. The small intestine exhibited perforation at a singular site, accompanied by adhesion of the intestines, mesentery, and omentum, alongside the existence of purulent fluid intermixed with fecal material within the abdominal cavity (Figure 1). 

Case 2

The man, aged 34 years old, also exhibited an average physique, displaying rigor mortis throughout the entirety of the body. The abdomen appeared moderately distended, with evidence of singed hairs on the trunk and right lower limb, along with marks indicating orthopedic traction pin insertion on the right lower limb. Diffuse areas of tattooing were present over the ventral aspect of the right hand, tip of the fingers of the right hand, posterolateral aspect of the right leg and knee, left forearm, left palm and fingers, fronto-medial aspect of the left leg, and anterior aspect of the middle part right side of the abdomen. 

**Figure 2:**
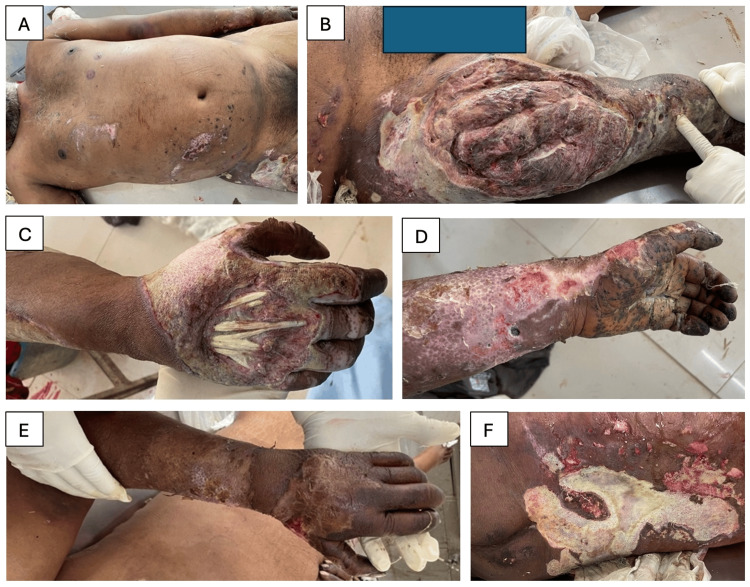
Autopsy photographs illustrating key findings in Case 2 A: Areas of tattooing over the right anterior aspect of the abdomen B: Bone-deep lacerated wound over the right thigh and traction pin insertion marks (therapeutic measure for fracture of the right femur) C: Soft tissue-deep lacerated wound over the dorsum of the right palm with exposed tendons D: Ventral aspect of the distal left forearm and left hand showing areas of tattooing, burns, and lacerations E: Dorsal aspect of the distal left forearm and left palm showing areas of burn F: Posterior aspect of the abdomen showing areas of burn

First- and second-degree infected burn areas involved the lower part of the posterior aspect of the chest and abdomen, both gluteal regions, lower part of the anterior aspect of the right side of the abdomen, lower part of the right arm, posterior and posteromedial aspect of the right forearm, lower part of the anterior aspect of the right side of the chest, lower part of the left forearm, left palm and fingers, dorsal aspect of the right palm and right wrist, anterior and posterolateral aspect of the left thigh, right knee, and right leg. Multiple soft tissue-deep infected lacerated wounds varying in sizes from 9×8 cm to 2×0.5 cm were present over the dorsal aspect of right palm, palmar aspect of the right ring finger and little finger, posterior aspect of the right forearm and right elbow, ventral aspect of the left palm, left index and little finger, and posteromedial aspect of the left palm. A bone-deep lacerated wound measuring 35×20 cm was present over the right thigh, with laceration of the soft tissue, blood vessels, and fracture of the upper part of the right femur bone (Figure 2).

## Discussion

Bomb explosion injuries in civilian settings present a unique and challenging medico-legal and public health concern, particularly when such incidents occur in densely populated urban marketplaces. The current case report details two civilian fatalities resulting from an accidental detonation of an explosive device in a vegetable market. The autopsy findings in both cases reflect the classical spectrum of blast injuries, thereby providing valuable insights into the medico-legal implications and pathophysiological outcomes of explosion-related trauma.

Primary blast injuries, as demonstrated in Case 1, involved the perforation of the small intestine (Table 1), indicating the susceptibility of gas-filled organs to rapid pressure changes. The presence of purulent fluid and fecal contamination in the abdominal cavity further highlights the risk of secondary infection and peritonitis, commonly observed in abdominal blast injuries. These injuries are often subtle and may be overlooked during initial examination, thereby underscoring the importance of thorough postmortem assessments in such cases.

**Table 1:** Salient characteristics of the cases

Case no.	Tattooing	Blast injuries				
		Primary	Secondary	Tertiary	Quaternary	Quinary
1	Yes	Perforation of the small intestine	Lacerated wounds	None	Singeing of hairs, first- and second-degree burn areas	None
2	Yes	None	Lacerated wounds, fracture of the right femur	None	Singeing of hairs, first- and second-degree burn areas	None

Both cases exhibited manifestations of secondary blast injuries (Table 1), which represent the most frequently documented occurrences in explosion-related events. These types of injuries arise from high-velocity projectiles, including shrapnel and debris, and were observed through the presence of numerous deep lacerations and, in the second case, a compound fracture of the femur. These observations are in alignment with prior research that underscores the prevalence of secondary injuries in blast events, particularly within urban civilian contexts, where fragmentation and debris from adjacent structures and objects are plentiful [8,12].

Quaternary injuries, which are defined by the presence of burns and singeing of hair, were observed in both subjects (Table 1). The widespread occurrence of first- and second-degree burns across the torso, limbs, and facial regions indicates the victims' close proximity to the blast epicenter and the substantial degree of thermal trauma sustained. It is particularly noteworthy that multiple burn sites in Case 2 exhibited signs of infection, thereby highlighting the complications that arise from open wounds in non-sterile environments. Even though burn injuries might not lead to immediate fatality, they considerably add to health complications and are an essential aspect of the prolonged impairments endured by blast incident survivors.

Interestingly, tertiary and quinary blast injuries were not identified in either case (Table 1). Tertiary injuries, typically resulting from bodily displacement or impact with solid structures, were not evident based on the absence of blunt trauma or internal hemorrhage unrelated to lacerations. Similarly, no signs of exposure to chemical, biological, or radiological agents were found, ruling out the presence of quinary injuries.

The sociodemographic profile of the victims aligns with established trends in India, where male individuals within the working age group are disproportionately affected by explosion-related trauma [8,9]. However, the predominance of female victims in this incident can be attributed to the localized occupational structure, with roadside female vendors being the primary occupants of the market area. This variation underscores the importance of context-specific risk assessments when analyzing blast injury patterns in civilian populations.

Autopsy findings such as diffuse tattooing, infected lacerations, and the severity of internal organ damage not only reflect the mechanical force of the explosion but also reiterate the urgent need for rapid response systems, public awareness, and disaster preparedness protocols tailored to urban civilian environments prone to such incidents. Furthermore, the forensic documentation of these injuries holds critical significance for legal investigations, victim identification, and compensation claims and in framing policies for urban safety and public health responses. The present case study highlights the necessity for integrating forensic medicine with trauma care systems to bridge existing gaps in blast injury management in resource-constrained settings.

## Conclusions

This case series pertaining to civilian casualties arising from inadvertent explosions elucidates the substantial hazards and repercussions linked to the improper handling of explosive substances within non-terrorist contexts. Such occurrences, frequently precipitated by carelessness or insufficient awareness, culminate in grave injuries and deaths, thereby accentuating the imperative for rigorous safety protocols and legislative measures while also highlighting the necessity of comprehending the underlying causes and enacting preventive interventions. Initiatives aimed at public awareness and educational programs possess the potential to markedly mitigate the likelihood of such events by informing individuals about the perils associated with the mishandling of explosive materials and the critical nature of adhering to established safety guidelines.
